# The Performance of the Intubation Difficulty Scale among Obese Parturients Undergoing Cesarean Section

**DOI:** 10.1155/2017/3075756

**Published:** 2017-01-26

**Authors:** Jatuporn Eiamcharoenwit, Napon Itthisompaiboon, Panita Limpawattana, Arunotai Siriussawakul

**Affiliations:** ^1^Anesthesiology Department, Prasat Neurological Institute, Bangkok, Thailand; ^2^Department of Anesthesiology, Maharat Nakhon Ratchasima Hospital, Nakhon Ratchasima, Thailand; ^3^Department of Internal Medicine, Faculty of Medicine, Khon Kaen University, Khon Kaen, Thailand; ^4^Department of Anesthesiology, Faculty of Medicine, Siriraj Hospital, Mahidol University, Bangkok, Thailand

## Abstract

*Background.* There have not yet been any studies to validate the intubation difficulty scale (IDS) in obese parturients. Objectives of this study were to determine the performance of the IDS in defining difficult intubation (DI) and to identify the optimal cutoff points of the IDS among obese parturients.* Methods.* This was a prospective observational study. Parturients with a body mass index ≥ 30 kg/m^2^ who underwent cesarean section utilizing endotracheal intubation were enrolled. The intubating performers were asked to assess the difficulty of endotracheal intubation and categorize it as easy, somewhat DI, and DI.* Main Results.* A total of 517 parturients were recruited with a mean BMI of 33.9 kg/m^2^. The incidence of some degree of DI was 14.5%. The area under the receiver operating characteristic curves of the IDS for detecting somewhat DI and DI was 1.0. The optimal cutoff point to define somewhat DI was ≥3 and DI was ≥5, which both had sensitivity and specificity of 100%.* Conclusions.* The IDS scoring is a good tool for defining DI among obese parturients. The IDS scores of ≥3 and ≥5 are the optimal cutoff points to define somewhat DI and DI, respectively.

## 1. Introduction

An unanticipated, difficult, tracheal intubation is a major cause of anesthetic-related maternal morbidity and mortality [1, 2]. In the case of obstetric anesthesia, difficult intubations have been reported to be 2.7% and failed intubations between 0.05% and 0.3% [3, 4]. Particularly in obese parturients impact of anesthetic management, there is an increase in metabolic rate, decreased functional residual capacity, and a shorter time available for intubation before desaturation develops [5, 6].

Unfortunately, there is no standard, accepted definition of difficult intubation among parturients. There is a wide variety of definitions of the term in medical literature. In 1997, Adnet et al. created a quantitative score for difficult intubation, known as the intubation difficulty scale (IDS) [7]. An IDS score is derived from an objective assessment of seven parameters which are known to be associated with difficult intubation. The sum of the points given for each parameter can be calculated immediately after an intubation, allowing it to be categorized as being an easy, slightly difficult, or moderate to severe difficult intubation. The IDS has been useful for evaluating predictive factors of difficult intubation. According to the definition proposed by The American Society of Anesthesiologists Task Force on Management of Difficult Airways, a difficult intubation is one in which a tracheal intubation requires multiple attempts, either in the presence or in the absence of tracheal pathology [8]. Other definitions of difficult intubation are associated with difficult laryngoscopy, which is only one of the seven parameters assessed by IDS. Cormack and Lehane defined four grades of laryngoscopic view, according to the structures that can be visualized. Intubation is easy for grade I, but it is slightly more difficult for grade II; grade III corresponds to severe intubation difficulties, while grade IV usually corresponds to an impossible intubation [9].

There have not yet been any studies assessing the difficulty of intubation for obese parturients using the IDS. The primary objective of this study was to determine the performance of the IDS in defining difficult intubation for obese parturients, based on a subjective assessment of the difficulty experienced. A secondary study objective was to identify the optimal cutoff points of the IDS for obese parturients.

## 2. Materials and Methods

### 2.1. Subject

This prospective observational study was approved by the Institutional Review Board of Maharat Nakhon Ratchasima Hospital, and informed consent was obtained from all parturients. The inclusion criteria were parturients aged > 18; parturients with a body mass index (BMI) of ≥30 kg/m^2^; and parturients who were undergoing a cesarean section with a conventional endotracheal intubation and/or regional anesthesia. The exclusion criteria were parturients who presented with an overtly difficult intubation, such as a facial fracture, a facial tumor, or cervical spine fractures; parturients who had a history of difficult intubation; parturients for whom a special technique for intubation was predetermined; and parturients who were overweight because of pathology such as ascites or an abdominal tumor.

### 2.2. Procedure

All parturients were assessed using a range of preoperative airway tests, namely, the neck circumference (NC), the sternomental distance (SMD), and the Modified Mallampati (MMT) tests. In the operating theatre, the anesthetic techniques employed were at the discretion of the anesthesiologists. The parturients were monitored routinely with an electrocardiogram, noninvasive arterial blood pressure, and pulse oximetry. A Macintosh blade number 3 could be used for the first laryngoscopy in each case. Parturients were arranged in a ramp or sniffing position. Preoxygenation was given using high-flow oxygen through a facemask for three to five minutes before anesthesia induction. Anesthesia was then induced with either intravenous sodium thiopental or propofol, and intravenous suxamethonium chloride was administered to facilitate endotracheal intubation. During loss of consciousness, cricoid pressure (also known as the Sellick maneuver) was applied to the patients. All tracheal intubations were performed by an anesthesiologist or a nurse anesthetist with at least two years' experience in anesthesia. Successful intubation was confirmed by bilateral auscultation of the lungs and capnography. Immediately after the endotracheal intubation, the operator was asked for a subjective assessment of the difficulty experienced by category: easy, somewhat difficult, and difficult. The points for each of the seven IDS parameters were collected, and the IDS score for each parturient was calculated by the principal investigator.

### 2.3. Intubation Difficulty Scale

The score is derived from seven variables. N1 represents the number of additional intubation attempts; the score is 0 for a first attempt. N2 represents the number of additional operators; the score is 0 if there is a successful intubation by the first operator. N3 is the number of alternative intubation techniques used; the score is 0 if there is no alternative intubation technique, but each alternative intubation technique utilized adds one point. N4 represents the laryngoscopic view, as defined by Cormack and Lehane: grade 1 = score 0, grade 2 = score 1, grade 3 = score 2, and grade 4 = score 3. N5 is the lifting force applied during laryngoscopy: score 0 if there is a normal lifting force, and score 1 if the force is considerable. N6 relates to the necessity to apply external laryngeal pressure to optimize glottic exposure: score 0 if there is no external pressure or only the Sellick maneuver is applied, and score 1 if external laryngeal pressure is used. N7 refers to vocal cord mobility: score 0 if they are abducted, and score 1 if they are adducted or not visible. The IDS score is the sum of N1 through N7 [7]. The total IDS score ranges from zero to infinity.

### 2.4. Statistical Analyses

#### 2.4.1. Sample Size Calculations

Sample size calculations were based on the primary objective of this study, which was the assessment of the performance of the IDS scores in the diagnosis of difficult intubation. Therefore, the areas under the ROC curve (AUC, utilizing the methodology of Hanley and McNeil) were used [10]. This method varies the sample size until a sufficiently small standard error (SE) of the area under the ROC curve is achieved. Because of the complexity of the formula, a web-based calculator (http://www.anaesthetist.com/mnm/stats/roc/#stderr) was used to determine the standard error. Finally, a sample size of 500 participants was found to be adequate and feasible to conduct the trial in clinical practice at an AUC of 0.9 and an SE of 0.03.

The parturient data collected was age, weight, height, BMI, ASA classification, indication for cesarean section, comorbid diseases, and the position for tracheal intubation. Data was presented as mean ± SD (range) or number (percent), as appropriate. The ROC curve was used to summarize the overall accuracy of the IDS score for identification of the intubation difficulty.

The ROC plot was obtained by calculating the sensitivity and specificity of every observed cutoff value and by plotting sensitivity against 1 − specificity. A value of 1.0 indicates perfect discrimination. The sensitivity, specificity, positive predictive value (PPV), negative predictive value (NPV), positive likelihood ratio, negative likelihood ratio, and Youden's index were obtained and compared among the cutoff values for difficult intubation. The data analyses were performed by using STATA version 10.0 (Stata Corp, College Station, TX, USA).

## 3. Results

### 3.1. Patient Characteristics

A total of 517 obese parturients were enrolled between October 2014 and January 2016. No parturients dropped out of the study. The demographic characteristics and preoperative airway assessment tests of the parturients are at Table 1. In the operating theatre, general anesthesia was mostly induced by thiopental (485, 93.8%) and propofol (32, 6.2%), and all parturients were intubated by administering suxamethonium chloride. The distributions of the Cormack-Lehane laryngoscopic view (LV) were 327 (63.2%), 171 (33.1%), 18 (3.5%), and 1 (0.2%) for grades 1, 2, 3, and 4, respectively.

### 3.2. The Performance of the IDS

**Figure 1:**
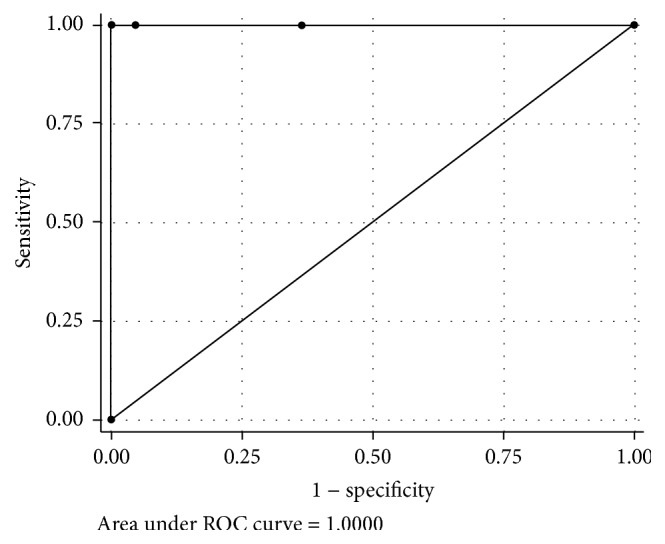
The area under the receiving operating characteristic (ROC) curve of the IDS in detection of somewhat difficult intubation.

**Figure 2:**
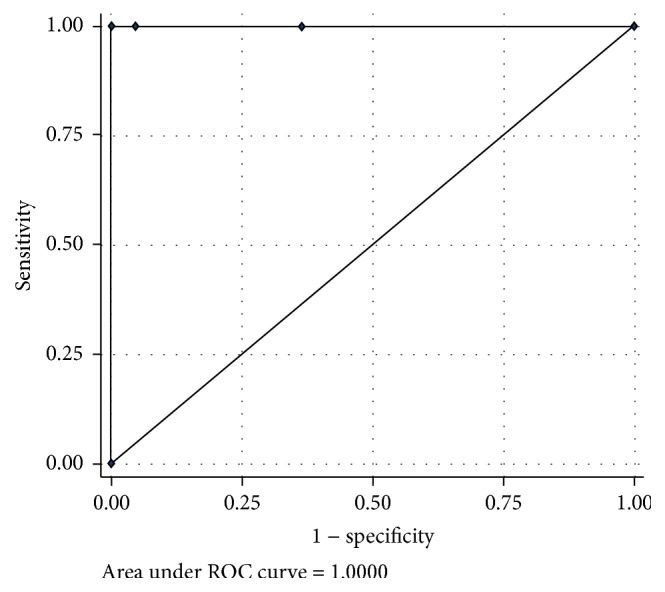
The area under the receiving operating characteristic (ROC) curve of the IDS in detection of difficult intubation.

The performance of the IDS and the operator-assessed subjective categories for detecting an easy intubation, a somewhat DI, and a DI among obese parturients are summarized at Tables 2 and 3. No failed intubation was reported in this study. The IDS for detecting somewhat DI and DI were represented by different cutoff points. The area under the receiving operating characteristic (ROC) curve of the somewhat DI and DI was 1.0, as shown in Figures 1 and 2. In this study, the optimal cutoff point to define somewhat DI was ≥3, and to define DI it was ≥5, as shown at Tables 4 and 5, thereby demonstrating high sensitivity and specificity.

### 3.3. The Time Taken to Finish Intubation

The average time between applying the laryngoscope until confirmation of a successful intubation was 20.5 ± 27.0 seconds, ranging from 10 seconds to 10 minutes. Six parturients (1.2%) required two attempts for intubation, while one parturient (0.2%) required five attempts. Three parturients (0.6%) achieved a successful intubation with a second operator, whereas one parturient (0.2%) achieved success with three operators.

**Figure 3:**
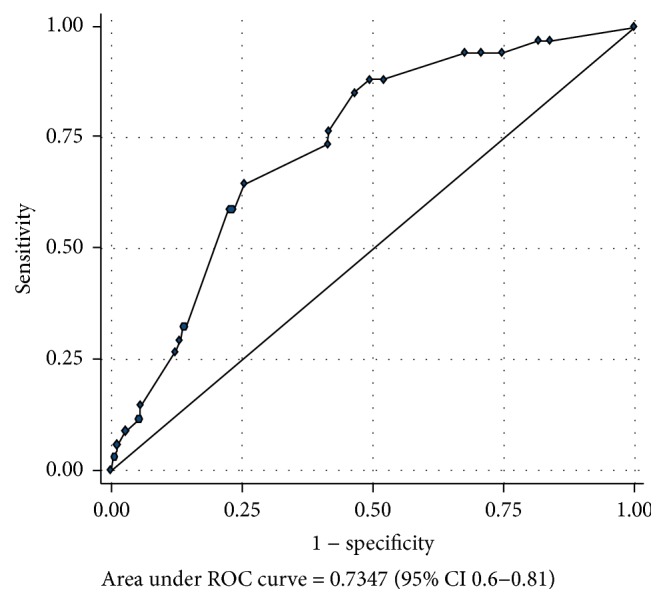
The area under the receiving operating characteristic (ROC) curve of the intubation time in detection of somewhat difficult intubation.

**Figure 4:**
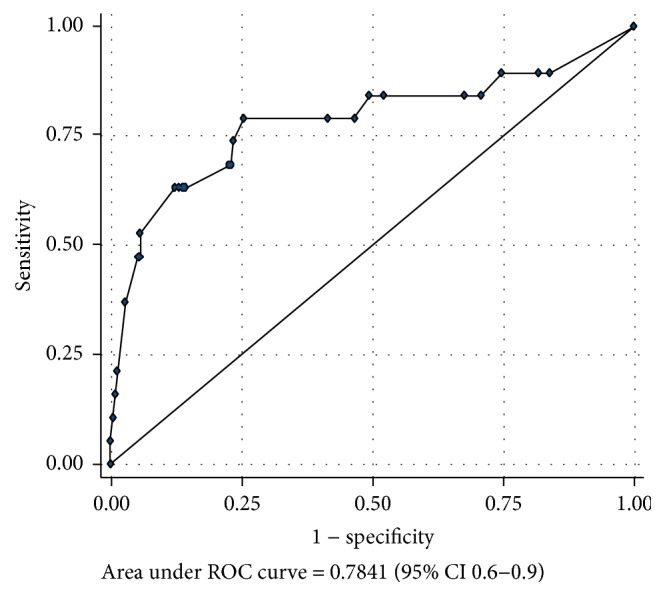
The area under the receiving operating characteristic (ROC) curve of the intubation time in detection of difficult intubation.

The performance of the intubation time for representing a somewhat DI and a DI, based on an ROC curve analysis, are summarized at Tables 6 and 7. The areas under the ROC curve of the IDS in detection of somewhat DI and DI were 0.73 (95% CI 0.6–0.81) and 0.78 (95% CI 0.6–0.9), as shown in Figures 3 and 4. The best cutoff points to complete an intubation for representing somewhat DI and DI were ≥22 seconds and ≥23 seconds, respectively, being the cutoff points with the highest area under the ROC curve.

## 4. Discussion

A WHO expert consultation recommended that a body mass index (BMI) cutoff point of ≥30 kg/m^2^ should be used as an international classification to determine obesity. However, this cutoff point was recognized as being inadequate as an indicator of obesity for many Asian populations because Asians are more likely to develop higher abdominal fat than white people at lower BMIs. Asian populations were identified as ≥23 kg/m^2^, representing increased risk, and ≥27.5 kg/m^2^ as representing high risk. Therefore, the cutoff point of obesity for Asians ranges between 23 and 27.5 kg/m^2^ [11]. Nevertheless, given that the incidence of difficult intubation among Thai obese patients is low [12], this study defined pregnant parturients with a BMI ≥ 30 mg/m^2^ as obese.

Previous studies have reported that parturients had an increased incidence of high Mallampati scores (classes 3 and 4) compared to their scores before getting pregnant, resulting in an increased risk of difficult laryngoscopy among the parturients. The changes in the Mallampati scores correlated with the weight gain occurring during pregnancy and fluid retention causing pharyngeal edema [13, 14]. Similarly, the results of this study found that women became overweight or obese after pregnancy. However, using the Mallampati classifications alone is not a good predictor of a difficult intubation in the case of obstetric patients [15].

We chose the intubation difficulty scale (IDS) to identify a difficult intubation for two reasons: the IDS is widely used by researchers and the IDS score is derived from seven variables. The grade of laryngoscopy seems to be an important component of the IDS score; however, poor visualization of the laryngoscopic view does not always mean there will be difficulty in intubation [16]. The incidence of difficult intubation in the parturients ranged between 3.4% and 8.75%, depending on the criteria used to define it [15, 17, 18].

We found a 14.5% incidence of some degree of difficult intubation in this study, and there were no failures in the tracheal intubations. The incidence of somewhat DI was 10.8% and of DI was 3.7%. A previous study in Thailand found that the IDS had good reliability in defining DI among obese patients, that a score of 2 or higher was an optimal cutoff point to indicate somewhat DI, and that a score of 5 or higher was an optimal cutoff point to indicate DI [12]. By comparison, our study found that a higher IDS score (≥3) was an optimal cutoff point for defining somewhat DI, but an IDS score of ≥5 was still an optimal cutoff point for defining DI among obese parturients. The results were different because of various factors, such as differences in the anatomic features of obese patients and obese parturient, cricoid pressure application, head position, degree of muscle relaxation, and the type or size of the laryngoscope blade used. Nevertheless, the results of both studies confirmed that an IDS score of ≥5 is an optimal cutoff point for defining DI.

The time necessary to finish an intubation was related to the definition of a difficult intubation formulated by the American Society of Anesthesiologists in 1993. A difficult intubation was defined as a tracheal intubation that took longer than ten minutes [19]. The results of the presented study showed an average time between applying the laryngoscope until confirmation of a successful intubation of 20.5 ± 27.0 seconds, ranging from 10 seconds to 10 minutes. The parturient that required ten minutes for intubation needed five attempts by three operators, the last of whom employed an alternative technique using a McCoy laryngoscope.

The best cutoff points to complete an intubation for detecting somewhat DI and DI were ≥22 seconds and ≥23 seconds, respectively, which demonstrated a moderate to good sensitivity and specificity. The performance of the endotracheal times to represent somewhat DI and DI was similar to the times taken for intubation. These results were different from the definition by The American Society of Anesthesiologists task force. Given the results of the presented study, the use of the time to achieve a successful intubation was found to be an ineffective way of communicating somewhat DI and DI in clinical practice. The factors affecting the time to successful intubation include, for example, operator skills, various techniques, and the degree of muscle relaxation.

The American Society of Anesthesiologists task force on the management of difficult airways recommended that an airway history should be conducted before the initiation of anesthetic care and airway management in all patients. Examination of previous anesthetic records may yield useful information about airway management [8]. We recommend that the IDS should be evaluated and recorded in patients' records in order to provide information on the endotracheal intubation. An IDS ≥ 5 should alert anesthetic personnel to prepare a strategy for difficult intubation. In particular, trainee or inexperienced anesthetic personnel should be aware of the possibility of a somewhat DI when an IDS of 3 or 4 is documented.

However, our study had some limitations. To increase its accuracy, the study should employ similar techniques of tracheal intubation, cricoid pressure application, head position, and degree of muscle relaxation. Additionally, this study was conducted at a tertiary hospital, which meant that the obese parturients were taken care of by physicians with significant experience. The factor that defines somewhat DI, DI, and time to completion of an intubation may be related to operator experience and the consequential prompt handling of a difficult airway situation. Therefore, the IDS score should be validated in another setting to confirm its accuracy. Nevertheless, our results should be applicable in other tertiary care settings.

In conclusion, the IDS score is a good tool to define difficult intubation among obese parturients. We prefer using an IDS score of ≥3 as an optimal cutoff point to define somewhat DI and an IDS score of ≥5 as an optimal cutoff point to define DI because of their high specificity and sensitivity. The intubation time is inappropriate to determine the degree of difficult intubation.

## Figures and Tables

**Table 1:** Demographic characteristics of parturients (*n* = 517).

Variables	Mean ± SD or number (%)
Age, years	29.2 ± 6.0
Body mass index, kg/m^2^	33.9 ± 3.7
Prepregnancy body mass index, kg/m^2^	27.8 ± 4.4
ASA classification	
II, III, IV	297 (57.4), 216 (41.8), 4 (0.8)
Indication of surgery	
Previous caesarean section	174 (33.7)
Cephalopelvic disproportion	189 (36.6)
Preeclampsia	57 (11.0)
Nonreassuring fetal status	15 (2.9)
Fetal macrosomia	10 (1.9)
Others	72 (13.9)
Airway assessment tests	
Neck circumference in sitting position, cm.	37.2 ± 3.0
Neck circumference in supine position, cm.	38.0 ± 3.0
Sternomental distance, cm.	15.9 ± 1.9
Modified Mallampati Test	
Classes I, II	65 (12.6), 288 (55.7)
Classes III, IV	155 (30.0), 9 (1.7)
Positions	
Sniffing	506 (97.9)
Ramp	11 (2.1)

**Table 2:** Scores of the intubation difficulty scale (IDS).

IDS score	Number (%)
0	295 (57.1)
1	147 (28.4)
2	22 (4.3)
3	26 (5.0)
4	8 (1.5)
5	6 (1.2)
6	9 (1.7)
7	3 (0.6)
14	1 (0.2)

**Table 3:** Operator-assessed subjective categorical impression of difficult tracheal intubation.

Operator-assessed subjective categories of difficult intubation	Number (%)
Easy	442 (85.5)
Somewhat difficult	56 (10.8)
Difficult	19 (3.7)

**Table 4:** The performance of the IDS among obese pregnant patients for detecting somewhat difficult intubation based on ROC curve analysis.

Cutoff point	Sensitivity (%)	Specificity (%)	PPV	NPV	LR+	LR−	AUC	Youden's index
≥1	100	63.6	16.7	100	2.75	0	0.82	0.64
≥2	100	95.3	60.7	100	21.1	0	0.98	0.95
≥3	100	100	100	100	—	0	1.0	1.0
≥4	0	100	100	94.7	—	1.0	0.62	0

*Note*. PPV; positive predictive value, NPV; negative predictive value, LR+; positive likelihood ratio, LR−; negative likelihood ratio, AUC; area under the ROC curve, Youden's index; *J* = sensitivity + Specificity − 1 (its value ranges from 0 to 1; a value of 1 indicates that there are no false positives or false negatives; i.e., the test is perfect).

**Table 5:** The performance of the IDS among obese pregnant patients for detecting difficult intubation based on ROC curve analysis.

Cutoff point	Sensitivity (%)	Specificity (%)	PPV	NPV	LR+	LR−	AUC	Youden's index
≥1	100	63.6	10.1	100	2.75	0	0.82	0.64
≥2	100	91.7	46.3	100	12.0	0	0.96	0.95
≥5	100	100	100	100	—	0	1.0	1.0
≥6	68.4	100	100	98.7	—	0.32	0.84	0.21

*Note*. PPV; positive predictive value, NPV; negative predictive value, LR+; positive likelihood ratio, LR−; negative likelihood ratio, AUC; area under the ROC curve, Youden's index; *J* = sensitivity + specificity − 1 (its value ranges from 0 to 1; a value of 1 indicates that there are no false positives or false negatives; i.e., the test is perfect).

**Table 6:** The performance of the endotracheal time among obese pregnant patients for detecting somewhat difficult intubation, based on ROC curve analysis.

Cutoff point (sec)	Sensitivity (%)	Specificity (%)	PPV	NPV	LR+	LR−	AUC	Youden's index
≥20	73.5	58.6	11.5	96.8	1.78	0.45	0.66	0.32
≥21	64.7	74.4	15.6	96.6	2.52	0.48	0.7	0.39
≥22	64.7	74.6	15.7	96.6	2.54	0.47	0.73	0.39
≥23	58.8	76.5	15.5	96.2	2.5	0.54	0.68	0.35

*Note*. PPV; positive predictive value, NPV; negative predictive value, LR+; positive likelihood ratio, LR−; negative likelihood ratio, AUC; area under the ROC curve, Youden's index; *J* = sensitivity + specificity − 1 (its value ranges from 0 to 1; a value of 1 indicates that there are no false positives or false negatives; i.e., the test is perfect).

**Table 7:** The performance of the endotracheal time among obese pregnant patients for detecting difficult intubation based on ROC curve analysis.

Cutoff point (sec)	Sensitivity	Specificity	PPV	NPV	LR+	LR−	AUC	Youden's index
≥20	78.9	58.6	7.25	98.6	1.91	0.36	0.69	0.38
≥21	78.9	74.4	11.2	98.9	3.08	0.28	0.77	0.54
≥22	78.9	74.6	11.3	98.9	3.1	0.28	0.77	0.54
≥23	78.9	76.5	12.1	98.9	3.36	0.28	0.78	0.55
≥24	68.4	76.9	10.8	98.3	2.97	0.41	0.73	0.45

*Note*. PPV; positive predictive value, NPV; negative predictive value, LR+; positive likelihood ratio, LR−; negative likelihood ratio, AUC; area under the ROC curve, Youden's index; *J* = sensitivity + specificity − 1 (its value ranges from 0 to 1; a value of 1 indicates that there are no false positives or false negatives; i.e., the test is perfect).
